# Riboflavin- and chlorophyllin-based antimicrobial photoinactivation of *Brevundimonas* sp. ESA1 biofilms

**DOI:** 10.3389/fcimb.2022.1006723

**Published:** 2022-09-21

**Authors:** Alisa Gricajeva, Irina Buchovec, Lilija Kalėdienė, Kazimieras Badokas, Pranciškus Vitta

**Affiliations:** ^1^ Department of Microbiology and Biotechnology, Life Sciences Center, Institute of Biosciences, Vilnius University, Vilnius, Lithuania; ^2^ Institute of Photonics and Nanotechnology, Faculty of Physics, Vilnius University, Vilnius, Lithuania

**Keywords:** bacterial biofilms, antimicrobial photoinactivation, riboflavin, chlorophyllin, *Brevundimonas*

## Abstract

Some *Brevundimonas* spp. are globally emerging opportunistic pathogens that can be dangerous to individuals with underlying medical conditions and for those who are immunocompromised. Gram-negative *Brevundimonas* spp. can form resilient sessile biofilms and are found not only in different confined terrestrial settings (e.g., hospitals) but are also frequently detected in spacecraft which is inhabited by astronauts that can have altered immunity. Therefore, *Brevundimonas* spp. pose a serious health hazard in different environments, especially in its biofilm form. Conventional antimicrobials applied to disrupt, inactivate, or prevent biofilm formation have limited efficiency and applicability in different closed-loop systems. Therefore, new, effective, and safe biofilm control technologies are in high demand. The present work aimed to investigate antimicrobial photoinactivation (API) of *Brevundimonas* sp. ESA1 monocultural biofilms mediated by non-toxic, natural photosensitizers such as riboflavin (RF) and chlorophyllin (Chl) with an emphasis of this technology as an example to be safely used in closed-loop systems such as spacecraft. The present study showed that Chl-based API had a bactericidal effect on *Brevundimonas* sp. ESA1 biofilms at twice the lower irradiation doses than was needed when applying RF-based API. Long-term API based on RF and Chl using 450 nm low irradiance plate has also been studied in this work as a more practically applicable API method. The ability of *Brevundimonas* sp. ESA1 biofilms to reduce alamarBlue™ and regrowth analysis have revealed that after the applied photoinactivation, bacteria can enter a viable but non-culturable state with no ability to resuscitate in some cases.

## 1 Introduction

Naturally most of the bacteria are found living in a multicellular coordinated functional communities known as biofilms. Bacterial biofilms consist of bacterial cells encased in a self-produced extracellular polymeric substance (EPS) made of polysaccharides, proteins, and extracellular DNA (eDNA) (Flemming et al., 2016; Penesyan et al., 2021). EPS is the underlying physical factor determining the ability of bacterial cells to be more resistant to adverse external impacts than free-living cells (Hall-Stoodley et al., 2004; Reichhardt et al., 2014). Bacterial biofilms are common in different industrial settings, food facilities, water systems, bathrooms, laboratories, hospitals, and even spacecraft (Buchovec et al., 2020). Once established, biofilms become less susceptible to common antimicrobials such as antibiotics, chemical disinfectants, physical stress, and the human immune system (Mora et al., 2019; Muhammad et al., 2020; Ciofu et al., 2022). Therefore, biofilms are of special concern not only in confined areas such as hospitals, industrial food-associated premises on Earth, but also closed-loop spacecraft systems. Human-manned spacecraft is a unique environment encountering dangerous microbial contamination even though the spacecraft is assembled in a cleanroom (Checinska et al., 2015; Mora et al., 2016; Bashir et al., 2016; Nakajima et al., 2017). Spacecraft conditions such as space radiation, microgravity, and elevated carbon dioxide levels adversely affect health and the immune system of the astronauts. Therefore, microbial contamination, especially in the form of biofilms, can be very dangerous for the immunocompromised spacecraft crew members and the overall material integrity (Decelle and Taylor, 1976; Sobisch et al., 2019). The most abundant airborne and surface bacteria in spacecraft belong to *Staphylococcus* spp., *Bacillus* spp., *Enterococcus* spp., *Corynebacterium* spp. and *Propionibacterium* spp. (Borisov et al., 2003; La Duc et al., 2004a; Novikova et al., 2006; Venkateswaran et al., 2014; Checinska et al., 2015; Koskinen et al., 2017; Regberg et al., 2020). *Methylobacterium* spp., *Sphingomonas paucimobilis*, *Cupriavidus* spp., *Chryseobacterium* spp., and *Ralstonia* spp. are most frequently found in potable water systems (La Duc et al., 2004b; Wong et al., 2010; Mijnendonckx et al., 2013; Nakajima et al., 2017; Thompson et al., 2020). Most spacecraft bacteria are human-associated and play the main role in the formation and the diversity of spacecraft microbiota (Mora et al., 2016; Be et al., 2017; Zea et al., 2018).

However, according to recent findings, one of the emerging bacterial genera that is not so abundant but is also frequently detected in different spacecraft samples is *Brevundimonas* spp. (Kawamura et al., 2001; La Duc et al., 2004b; Li et al., 2004; Ghosh et al., 2010; Stieglmeier et al., 2012; Vornhagen et al., 2013) *Brevundimonas* spp. are non-fermenting Gram-negative bacteria that can form sessile biofilms, with some of the species being a cause of serious infections in individuals with underlying medical conditions (Vornhagen et al., 2013). The genus was established by Segers et al. (1994) when authors presented the re-classification of *Pseudomonas diminuta* and *Pseudomonas vesicularis* to *Brevundimonas diminuta* and *Brevundimonas vesicularis*, respectively (Segers et al., 1994). *B. diminuta* and *B. vesicularis* are concerned to be emerging global opportunistic pathogens due to recent findings of multiple infections caused by these species indicating that the genus may be a more widespread pathogen than it was hitherto thought. And apparently, infections caused by *Brevundimonas* spp. can be invasive and dangerous for people having chronic diseases or those who are immunocompromised (Han and Andrade, 2005; Ryan and Pembroke, 2018). Therefore, it is a matter of concern that the species of the genus are being constantly detected not only in different terrestrial facilities but also in spacecraft. In a varying abundance, *Brevundimonas* spp. was recovered from ISS-associated potable water samples at various stages of their purification, storage, and transport. Detection of *Brevundimonas* spp. in the water system of spacecraft means that it can survive in spacecraft essential systems, forming more resistant biofilm forms (La Duc et al., 2004b; Vornhagen et al., 2013). A study of the abundance and diversity of microbial bioburden in European spacecraft-associated clean rooms by molecular analysis revealed that *Brevundimonas* was among the most common (Stieglmeier et al., 2012).

Recently, *Brevundimonas* sp., among some other species, was determined to form biofilms that developed higher concentration of antibiotic resistant bacteria (ARB) under the disinfection pressure of chlorination and chloramination. This study indicated that biofilm detachment might become a cause of the movement of biofilm clusters with higher ARB concentration into water, thereby increasing the antibiotic resistance of bacteria in tap water (Zhang et al., 2019a). In a study of Low et al. (2016), as a genus of *Proteobacteria*, *Brevundimonas* was found to be resistant to eight antibiotics and was reported to contain tetracycline resistance genes (Miranda et al., 2003; Adelowo and Fagade, 2009). *Brevundimonas* spp. can exhibit resistance to heavy metals as well (Zhang et al., 2019b).

The most interesting finding about *Brevundimonas* is that it is found to be one of a few bacteria exhibiting high survival rates under simulated Martian conditions. Dartnell et al. (2010) have studied the survival responses of some novel psychrotolerant bacterial strains (isolated from the Antarctic Dry Valleys) to ionizing radiation while frozen at -79 °C, the temperature that is typical to Martian near-subsurface environment. Interestingly, one of the novel isolates of the Antarctic Dry Valleys was identified as *Brevundimonas* sp. MV.7 and was determined to be the most resistant to radiation. Experimental irradiation combined with previous radiation modelling indicated that *Brevundimonas* sp. MV.7 in 30 cm deep Martian dust could survive the space radiation for up to 100,000 years before having a 10^6^ population reduction (Dartnell et al., 2010).

Therefore, some of the *Brevundimonas* species, due to its multiple resilience should pose serious concern in some terrestrial and especially spacecraft environments. Since conventional antimicrobials applied to disrupt, inactivate, or prevent biofilm formation have limited efficiency and applicability in closed-loop systems like spacecraft, new, effective, environmentally friendly, and safe biofilm control technologies are in a high demand.

One of the potential alternatives that provide many significant advantages is antimicrobial photoinactivation (API) (also known as antimicrobial photodynamic therapy - aPDT) - a technology based on interaction between non-toxic photosensitizer (PS), molecular oxygen, and appropriate doses of visible light of a certain wavelength that excites the PS (St. Denis et al., 2011; Hamblin, 2016). Usually, after light excitation, the triplet-state of PS interacts with molecular oxygen, electron donors or acceptors and can produce reactive oxygen species (ROS), thereby triggering photo-oxidative reactions that initiate various cellular damages and destruction of microorganisms (Hu et al., 2018). Different investigators have confirmed that microorganisms, including bacteria, viruses, molds, and protozoa, whether *in vitro* or *in vivo*, can be killed by API treatment (Wainwright, 2004; Jori and Brown, 2004; Luksiene, 2005; Buchovec et al., 2016; Temba et al., 2016). One of the major advantages of API is that the resistance of bacteria to API is unlikely to occur and it can be safely used in closed-loop systems (Liu et al., 2015; Maisch, 2015; Kashef and Hamblin, 2017). For the inactivation of both planktonic and sessile biofilm forms of bacteria in spacecraft and other sensitive confined systems - a non-toxic, chemically pure and stable, non-bleaching, easy-to-produce, and water-soluble PSs should be used. Most of the natural PSs meet the above-listed criteria and are one of the safest options of PSs for spacecraft use that have been proposed to date. Currently, four main natural products have been applied for API: curcumin, riboflavin, perylenequinones (hypericin, hypocrellin), psoralens (Yin et al., 2014). Recently, chlorophyll derivatives, such as sodium chlorophyllin were shown to be effective as natural PSs as well (Luksiene et al., 2010; Buchovec et al., 2010; Buchovec et al., 2017; Krüger et al., 2019; Luksiene and Buchovec, 2019; Buchovec et al., 2022). This study focuses on natural promising PSs - riboflavin (RF) or vitamin B2 and chlorophyllin (Chl) that could be potentially used in closed-loop systems as non-toxic and harmless to humans and plants. Both PSs – RF and Chl - are known photoactive compounds used as food colorants having “Generally Recognized As Safe” (GRAS) status. Also, it is essential to note that both PSs, RF and Chl, are photoactive in the visible-light range and have absorption maximums at 440 and 402 nm, respectively (Buchovec et al., 2020). RF is a naturally occurring water-soluble compound and an essential human nutrient and is used as a food colorant E 101. It plays an important role in the metabolism of the cells and can be considered safe when administered to humans (Astanov et al., 2014; Sheraz et al., 2014). Chl is also water-soluble food colorant - sodium magnesium chlorophyllin (E 140 (ii)), which is extracted from different plants such as spinach, grass, dandelion, green cabbage, water hyacinth, and algae. Chl is a semi-synthetic porphyrin obtained from chlorophyll which generates ROS with antimicrobial activity after exposure to visible light (Buchovec et al., 2017). Nevertheless, studies and applications of the RF-based (RF-API) and Chl-based (Chl-API) API to control Gram-negative bacteria pathogens, especially their biofilms, remain scarce. Furthermore, no API, neither with natural nor with other PSs has been performed on *Brevundimonas* spp. that are a matter of concern in both terrestrial and spacecraft settings. The present study aims to investigate whether RF-based and Chl-based API can efficiently inactivate biofilms of *Brevundimonas* sp. ESA1.

## 2 Materials and methods

### 2.1 Photosensitizers

Non-copperized chlorophyllin sodium salt (Chl) (M_W_ = 684.9 g/mol) was obtained from Carl Roth (Germany). The aqueous stock solution of 0.15 mM Chl (pH = 6.8) was prepared by dissolving Chl in distilled water. Riboflavin (RF) (M_W_ = 376.36 g/mol)) was obtained from Sigma-Aldrich (USA). Aqueous stock solution of 0.11 mM RF (pH 6.2) was prepared according to the method of Mazzotta et al. (2014): RF was dissolved in distilled water using a magnetic stirrer at 25°С in the dark. Both PSs were filter-sterilized before use for the photoinactivation experiments. All working solutions were freshly prepared by diluting them with 0.01 M PBS buffer (pH 7.4) (Carl Roth, Germany) on the day of use.

### 2.2 Light source

**Figure 1:**
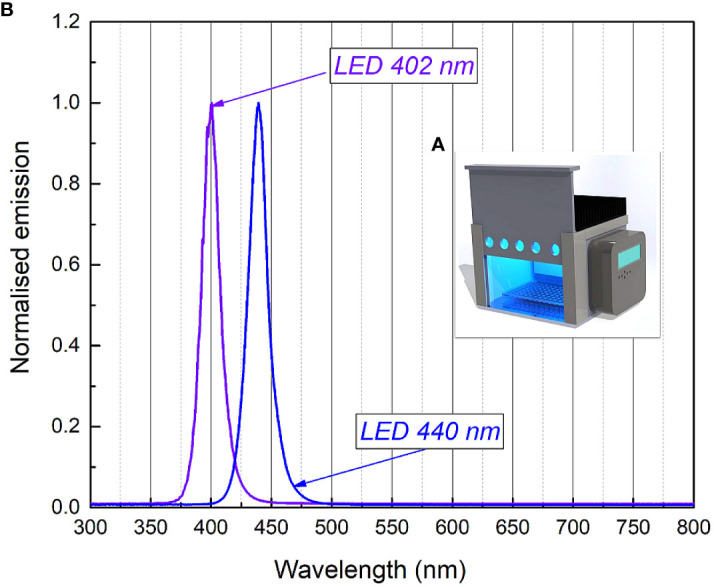
3D render picture **(A)** of the developed illumination system (the irradiation box) and normalized emission spectra of the 402 nm (Kingbright KTDS-3534UV405B), 440 nm (Osram GD QSSPA1.14) LEDs installed into the illumination system **(B)**.

The LED-based light source (an irradiation box) (**Figure 1A**) for RF-API and Chl-API was developed at the Institute of Photonics and Nanotechnology of Vilnius University (VU). Two types of LEDs (402 nm and 440 nm) with emission peaks near the maximum absorption of RF and Chl (Buchovec et al., 2022) were used (**Figure 1B**).

The light irradiance at the surface of the samples reached 5, 20 and 25 mW/cm^2^. The irradiation dose was calculated as irradiance multiplied by irradiation time. The sample exposure time was adjusted according to the equation:


E=P×t

where *E* is the energy density (dose) in J/cm^2^, *P* is the irradiance (light flux density) in mW/cm^2^, and *t* is the time in seconds. The exemplary irradiation dose calculations are shown in **Table 1**.

**Table 1:** Irradiation doses used in experiments.

Time (min)	Irradiation dose (J/cm^2^)		
	402 nm/440 nmIrradiance 5 mW/cm^2^	402 nmIrradiance 20 mW/cm^2^	440 nmIrradiance 25 mW/cm^2^
2	0.6	n	n
5	1.5	n	n
10	3	n	n
15	4.5	n	n
20	6	n	n
25	7.5	n	n
30	9	36	45
35	10.5	n	n
45	13.5	n	n
55	16.5	n	n
60	n	72	90
65	19.5	n	n
75	22.5	n	n
90	27	108	135
120	36	144	180
150	n	180	n
180	n	216	n

n-was not used.

**Figure 2:**
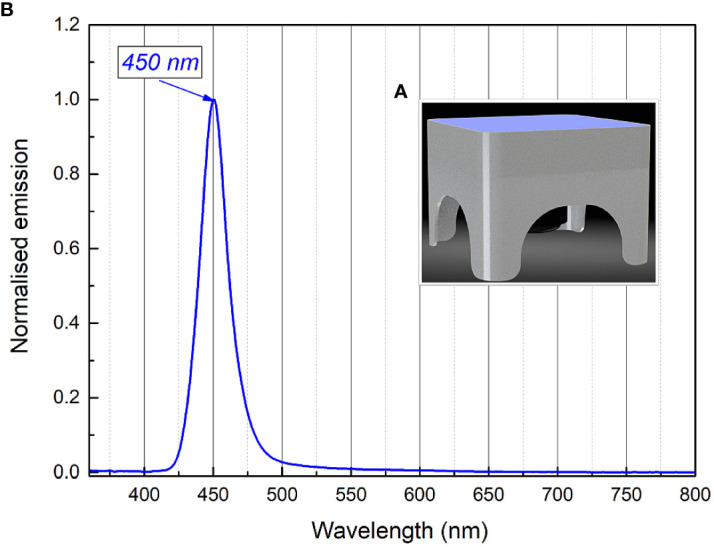
Constant irradiance plate 3D model **(A)**, relative irradiance spectrum **(B)**.

The light source used for long-term irradiation of biofilms was developed and constructed as a Constant Irradiation Plate (CIP; developed in the Institute of Photonics and Nanotechnology, VU) with 1.4 mW/cm^2^ uniform irradiance by LEDs peaked at 450 nm (**Figure 2**). The CIP is a multicolor micro-controller controlled LED source for upward irradiation. The device has an internal temperature feed-back circuit to maintain irradiation stability under long-term experiments. CIP was calibrated before the experiments with a spectro-radiometer (Avantes AvaSpec-ULS2048LTEC with AvaSphere-50-LS-HAL-CAL).

### 2.3 Spectrophotometric measurements of RF and Chl

Aqueous solutions of 0.011 mM RF (pH 7.4) and 0.015 mM Chl (pH 7.4) were illuminated with a LED-based light source (irradiation box) for analysis of the photostability of the PSs. The changes in the absorption spectra of RF and Chl were investigated after LED illumination at 440 nm and 402 nm, respectively. For this purpose, 200 µL of the PSs were transferred to sterile flat-bottom 96-well microtiter plates (MtP) and exposed to 5 mW/cm^2^ irradiance. After each irradiation dose applied separately, the samples (3 mL) were collected into cuvettes and used for spectrophotometric measurements.

The absorption spectra of both PSs solutions were recorded by means of a LAMBDA 950 UV-VIS-NIR spectrophotometer (PerkinElmer, USA) in spectral range of 300-600 nm. Polymethyl methacrylate cuvettes of 1 cm optical thickness were used for the measurements. All measurements were performed at 20 ± 2°C.

### 2.4 Bacterial genus confirmation by 16S rDNA analysis

Bacterial strain that was used as a model organism for RF-API and Chl-API studies was obtained from the collection of microorganisms of the Department of Microbiology and Biotechnology, Institute of Biosciences, Life Sciences Center, VU. For the confirmation of the genus of the strain, DNA sequence of the bacteria coding for 16S rRNA was amplified as described previously (Gricajeva et al., 2016) using 27F (5′-GAG AGT TTG ATC CTG GCT CAG-3′) and 1495R (5′-CTA CGG CTA CCT TGT TAC GA-3′) universal primers (METABION, Germany). Purified amplicon was sequenced at VU Life Sciences Center, Institute of Biotechnology (Lithuania). NCBI Basic Local Alignment Search Tool (BLASTn) (Altschul et al., 1990) was used for database similarity searches.

### 2.5 Culture conditions of planktonic bacterial cells

Bacteria were grown in Luria Bertani (LB) (Carl Roth, Germany) broth at 37°C under constant 180 rpm shaking (IKA, Germany) overnight. The overnight culture was inoculated to a fresh LB media and grown under the same conditions until optical density (OD) of 0.22 at 600 nm (OD_600_) corresponding to the concentration of 10^9^ CFU/mL was reached. Bacterial cells were harvested by centrifugation (5 min, 5000 × *g*), resuspended in 0.01 M PBS (pH 7.4) and immediately used for the RF-API and Chl-API experiments.

### 2.6 Photoinactivation of planktonic bacteria

For the assay of antimicrobial inactivation of free-floating (planktonic) *Brevundimonas* sp. ESA1, bacterial cells of the strain were resuspended to a final concentration of 10^7^ CFU/mL in 0.011 mM RF (pH 7.4) or 0.015 mM Chl (pH 7.4) in the dark. Solution consisting of bacterial suspension in 0.01 M PBS (pH 7.4) was used as a control. Aliquots of 200 μL of prepared mixtures of bacterial suspensions with appropriate PSs (RF or Chl, pH 7.4) or 0.01 M PBS (pH 7.4) were pipetted into sterile flat-bottom 96-well polystyrene MtP and exposed to different irradiation doses (**Table 1**). The light source (**Figure 1A**) used for the photoinactivation experiments consisted of a LED array (λ = 440 nm for RF and λ = 405 nm for Chl) with an intensity of 5 mW/cm^2^ at a distance of approximately 7 cm. Following irradiation, at each sampling step, withdrawn bacterial suspensions were appropriately diluted, spread on LB agar plates and incubated at 37°C for 16-36 h. Residual bacterial cell viability was determined by counting CFU. The numbers of surviving bacteria (CFU/mL) were transformed to log_10_ scale. CFU counts of the bacterial cells that were incubated with RF, Chl or PBS in the dark (dark controls) were also determined.

### 2.7 Monocultural bacterial biofilm formation in microtiter plates

For the monocultural biofilm formation bacterial strain was grown as it was described in Section 2.5 until OD_600_ corresponding to the concentration of 10^8^ CFU/mL. Then 100 µL of the suspension was pipetted into sterile flat-bottom 96-well polystyrene MtP wells. MtPs with the required number of wells filled with bacterial suspension were statically incubated 20 h at 37°C. Biofilms that formed in the wells of MtP were washed three times with 0.01 M PBS (pH 7.4) in order to remove residual planktonic cells. Biofilms were further used for the API (Section 2.8). Formation of *Brevundimonas* sp. ESA1 biofilms in the MtP wells was verified by staining with 0.1% solution of crystal violet (Merritt et al., 2005; Trotonda et al., 2008).

### 2.8 Photoinactivation of biofilm

*Brevundimonas* sp. ESA1 biofilms were formed in MtPs as it was described in Section 2.7. For the API, the wells of the 96-well MtPs containing the biofilm were filled with 0.011 mM RF (pH 7.4), 0.015 mM Chl (pH 7.4) or 0.01 M PBS (pH 7.4) by adding 200 µL of the appropriate solution. Then biofilms were immediately placed into irradiation boxes and irradiated with 440 nm at 25 mW/cm^2^ or 402 nm at 20 mW/cm^2^ for the photoactivation of RF and Chl, respectively. The control samples were also illuminated with the same wavelength of light. Biofilms were exposed to different illumination doses (**Table 1**). At each step of sampling, bacterial biofilms were mechanically detached from the MtP well walls, vigorously vortexed and diluted for the further viability determination by counting CFU on LB agar plates which after the spreading of samples were incubated at 37°C for 16-36 h. The numbers of surviving bacteria (CFU/mL) were transformed to log_10_ scale. CFU counts of *Brevundimonas* sp. ESA1 biofilm-forming cells that were incubated with RF/Chl or without RF/Chl in the dark were also determined.

### 2.9 Viability assay of photoinactivated biofilms using alamarBlue™ by fluorescence

Following photoinactivation, additionally to residual CFU count determination after RF-API and Chl-API, viability, and metabolic function of *Brevundimonas* sp. ESA1 biofilms was quantitatively analyzed by evaluating their ability to reduce resazurin-based compound alamarBlue™ (Invitrogen, USA). Non-toxic reagent alamarBlue™ is used as an indicator of cellular reducing environment or cell viability and death. The reagent is modified in reducing conditions that are characteristic to viable cells and becomes detectable due to its subsequently occurring color change or/and high fluorescence. Dead or non-viable cells are not able to change the color of alamarBlue™ (Rampersad, 2012).

In the current experiment *Brevundimonas* sp. ESA1 biofilms after RF-API and Chl-API that caused ≥3 log_10_ reduction (and corresponding dark controls) were mechanically detached, vortexed and added to alamarBlue™. The volume ratio of alamarBlue™ and disrupted biofilms was 1:10. Samples were incubated 3 h at 37°C (according to the manufacturer’s recommendations) and then fluorescence changes were measured every hour (in total for 11 h) using a plate reader (Thermo Fisher Scientific Verioscan Flash, USA). Fluorescence changes were read using excitation at 560 nm and emission at 590 nm.

Percentage reduction of alamarBlue™ by fluorescence indicating cell viability or death of all RF-API and Chl-API tested groups was determined by using the equation: **% reduction of alamarBlue™** =


, $$\frac{FI\;590\;of\;test\;agent-FI\;590\;of\;untreated\;control}{FI\;590\;of\;100\%\;reduced\;alamarBlue-FI\;of\;untreated\;control}\;\times 100\%$$

where FI 590 is fluorescence intensity at 590 nm emission (excitation at 560 nm)

### 2.10 Long-term RF- and Chl-API of *Brevundimonas* sp. ESA1 biofilms

Long-term RF-based and Chl-based API experiments were performed to test the photoinactivation efficacy of *Brevundimonas* sp. ESA1 biofilm by using CIP that emits 450 nm blue light. For the long-term RF-API and Chl-API experiments, *Brevundimonas* sp. ESA1 biofilms were formed as described previously (Section 2.7). Wells with biofilms filled with 200 µL of 0.015 mM Chl, 0.011 mM RF and 0.01 M PBS solutions were illuminated with 450 nm light on the CIP (**Figure 2A**) at 1.4 mW/cm^2^ irradiance for 28 h (to achieve irradiation dose of 141.1 J/cm^2^) for RF-API and 20 h (to achieve irradiation dose of 100.8 J/cm^2^) for Chl-API. Irradiation doses using CIP were chosen to correspond to those that caused ≥3 log_10_ reduction of CFU of biofilms using irradiation boxes. The irradiation dose of long-term Chl-API was higher than using the irradiation box due to suboptimal Chl photoactivation wavelength emitted by CIP. Results of long-term irradiation were evaluated by determining CFU counts on LB plates and the ability of the photoinactivated biofilm cells to reduce alamarBlue™, as described previously (Sections 2.8 and 2.9).

### 2.11 *Brevundimonas* sp. ESA1 re-growth after photoinactivation

For the evaluation of *Brevundimonas* sp. ESA1 biofilm-forming cell’s capability to resuscitate and re-grow after API, mechanically detached and vortexed biofilms were inoculated into liquid LB media and incubated for 72 h at 37°C under constant 180 rpm shaking. During the incubation, OD_600_ of the bacteria was measured once in 24 h. Re-growth capability of the dark controls (bacterial biofilms incubated with and without RF/Chl in the dark) of *Brevundimonas* sp. ESA1 were evaluated in the same way as for the irradiated bacteria.

### 2.12 Scanning electron microscopy of biofilms

The effect of RF-API and Chl-API on the morphology of *Brevundimonas* sp. ESA1 biofilm were investigated by scanning electron microscopy (SEM) (CamScan Apollo 300, Cambridge, UK). For the SEM analysis biofilms were grown in MtPs as it is described in Section 2.7. After the RF-API and Chl-API treatment (as described in Section 2.8) at 25 mW/cm^2^ for 95 min and 20 mW/cm^2^ for 60 min (irradiation conditions under which 3 log_10_ reduction was achieved), respectively, irradiated biofilms and dark controls were mechanically detached by scraping them of the MtP well walls by pipette tip, then 10 µl of each sample was transferred on a SEM specimen stub covered with copper foil tape, air-dried at room temperature and coated with 50 nm gold layer using Q150T ES sputter coater (Quorum Technologies, Lewes, England). The scanning was performed using an electron beam with an accelerating voltage of 20 kV.

### 2.13 Statistical analysis

All experiments were performed at least three times (independently) and the results were reported providing mean ± SD. Students t-test and one-way ANOVA statistical tests were applied. Statistically significant differences among groups were considered when p ≤ 0.05. Graph construction was performed with Origin Pro 8.1 (OriginLab Corporation, USA) and GraphPad Prism V. 6 (GraphPad Software, USA) software. Statistical analysis was performed with GraphPad Prism V. 6 software.

## 3 Results

### 3.1 RF and Chl photostability study

Before the API of *Brevundimonas* sp. ESA1, the photostability of the aqueous solutions of 0.011 mM RF (pH 6.4) and 0.015 mM Chl (pH 6.8) was tested. It is known that RF absorbance spectra have four maximums: 223, 267, 373, 444 nm (Astanov et al., 2014). Therefore, the LEDs of 440 nm (irradiation box, Section 2.2) were used in experiments for the optimal excitation of RF. Chl is widely known as a water-soluble photoactive compound with the main absorption maximum at about 405 nm (Buchovec et al., 2017). Therefore, 402 nm LEDs of the irradiation box were used in experiments for the optimal excitation of Chl.

**Figure 3:**
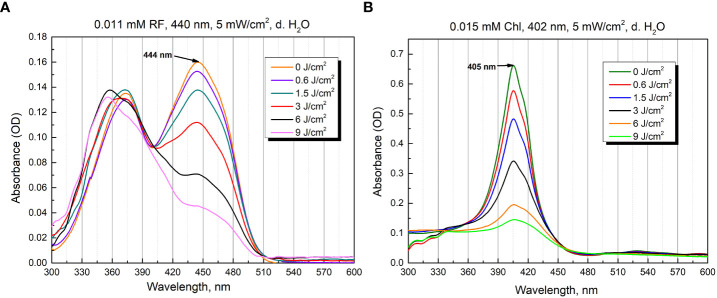
Photostability as optical absorbance spectra of 0.011 mM RF **(A)** and 0.015 mM Chl **(B)** after treatment of different irradiation doses (5 mW/cm^2^ irradiance).

Both PSs are known to exhibit optical absorbance reduction after the activation by light. These changes show the activation dependence on excitation dose and can be used to compare the irradiation efficiency by different spectral components. We primarily studied the absorption characteristics of RF and Chl after illumination with an optimal excitation wavelength of 440 nm and 402 nm, respectively (**Figure 3**).

The aqueous solutions of RF are sensitive to light and degraded to various photoproducts: formylmethylflavin, lumichrome, lumiflavin, carboxymethylflavin, 2,3-butanedione, a β-keto acid and a diketo compound. The type of the photoproduct depends on the solvent, pH, buffer type, concentration, oxygen content, light intensity, and wavelengths used (Sheraz et al., 2014). **Figure 3A** illustrates the spectra of 0.011 mM RF (pH 6.4) after blue light irradiation at 5 mW/cm^2^ for several durations (0 - 30 min, corresponding to 0-9 J/cm^2^ irradiance doses). As shown in **Figure 3A**, the absorbance of 0.011 mM RF at 373 and 444 nm was dramatically decreased after 440 nm irradiation (9 J/cm^2^). The photodegradation experiments helped assess the level of RF stability and revealed its photodegradation products after certain illumination exposures. Studies have shown that 0.011 mM RF (pH 6.4) photodegraded and probable formation of lumichrome photoproduct is observed at 9 J/cm^2^ (440 nm) exposure, according to the previous studies (Buchovec et al., 2022).

**Figure 3B** shows the spectra recorded after illumination of 0.015 mM Chl solution (pH 6.8) in distilled water at 5 mW/cm^2^ for different time periods (0-30 min, which corresponds to 0-9 J/cm^2^ irradiance doses). The illumination at 402 nm significantly diminished the peak magnitude after 30 min (9 J/cm^2^).

Although PSs were photobleached after 30 min of irradiation, antimicrobial effect can still be effectively implemented. In the case of RF, photoproducts, which can serve as PSs, are produced. Additionally, 440 nm light itself can have antibacterial effect as well. In the case of Chl, after its photoexcitation ROS that can cause significant damage are produced, and further, when Chl photodegrades, the ROS produced by 402 nm itself can have an antibacterial effect (Gwynne and Gallagher, 2018; Buchovec et al., 2022; Hadi et al., 2020).

### 3.2 RF- and Chl-API of planktonic bacteria and biofilms

The main objective of the study was to determine the bactericidal effect of RF-based and Chl-based API on *Brevundimonas* sp. ESA1 monocultural biofilm, although planktonic cells were also studied. RF and Chl were used to inactivate planktonic cells and biofilm in combination with 440 nm and 402 nm lights, respectively. Moreover, *Brevundimonas* sp. ESA1 biofilms were subjected to RF-API and Chl-API using a lower irradiance plate emitting 450 nm light. Partial sequence of 16S rRNA gene of the microorganisms used as a model in this study was deposited in GeneBank under the accession number ON237360.

#### 3.2.1 Photoinactivation using 402 and 440 nm LED irradiance box

**Figure 4:**
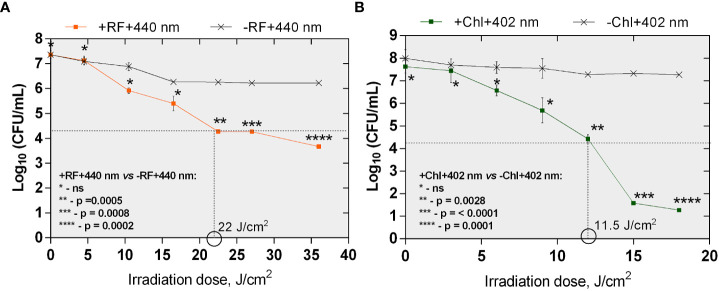
The effect of RF-API **(A)** and Chl-API **(B)** on *Brevundimonas* sp. ESA1 free-floating (planktonic) cells. Horizontal and vertical dashed lines shown in the graphs indicate the minimal irradiation doses thet were required to achieve a 3 log_10_ reduction of CFU counts of the planktonic cells. +RF +440 nm: irradiated using RF, -RF +440 nm: irradiated not using RF, +Chl +402 nm: irradiated using Chl, -Chl +402 nm: irradiated not using Chl; ns – non-significant. Error bars of some points are too small to be visible.

Bactericidal effect of API was defined as ≥3 log_10_ (99.9%) reduction in count of CFU according to the National Committee for Clinical Laboratory (NCCLS), M26-A standard (Thornsberry, 1983; CLSI, 1999). The minimal required dose of irradiation for the achievement of reduction of ≥3 log_10_ in CFU counts of planktonic cells after RF-API was determined to be 22 J/cm^2^. Inactivation causing ≥3 log_10_ reduction in CFU counts after Chl-API required almost twice lower irradiation dose - only 11.5 J/cm^2^ (**Figures 4A, B**).

During RF-API and Chl-API, respective samples of dark controls (samples that were incubated in the dark during/in parallel to API illuminations) were collected and analyzed by determining CFU counts as well. Respective dark controls with and without PSs (RF or Chl) during the time of its incubation parallel to the illumination experiments did not change significantly and fluctuated within appropriate limits (**Table 2**).

**Table 2:** CFU counts of dark controls (reported in log_10_ scale) of the planktonic cells.

	Planktonic bacteria growth mode											
		-RF-440 nm ^**^ (CFU/mL)						+RF*-*440 nm^***^ (CFU/mL)					
Incubation time, min	15	35	55	75	90	120	15	35	55	75	90	120
	6.8± 0.08*	6.9± 0.07*	6.6± 0.1*	6.5± 0.1*	6.5± 0.1*	6.5± 0.05*	6.9± 0.1*	6.8± 0.1*	6.7± 0.03*	6.5± 0.07*	6.5± 0.1*	6.4± 0.07*
	-Chl-402 nm^*^ (CFU/mL)						+Chl -402 nm^**^ (CFU/mL)					
Incubation time, min	10	20	30	40	50	60	10	20	30	40	50	60
	7.67± 0.1*	7.63± 0.3*	7.7± 0.08*	7.7± 0.2*	7.43± 0.1*	7.42± 0.02*	7.58± 0.3*	7.5 ± 0.2*	7.52± 0.1*	7.23± 0.2*	7.42± 0.01*	7.48± 0.07*

* - +RF+440 nm vs -RF-440 and +RF-440 nm or +Chl+402 nm vs –Chl-402 nm and +Chl-402 nm ANOVA test p value <0.05,

** - dark control without RF or Chl, respectively; *** - dark control with RF or Chl, respectively; n – was not evaluated.

**Figure 5:**
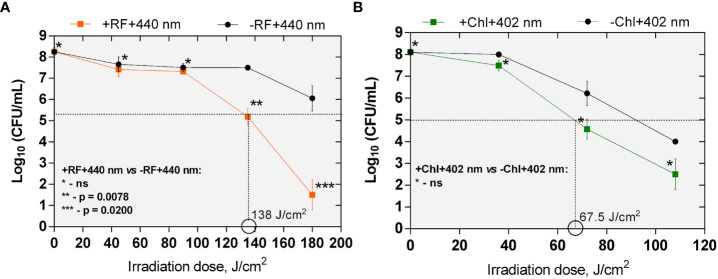
The effect of RF- **(A)** and Chl-API **(B)** on *Brevundimonas* sp. ESA1 biofilm. Horizontal and vertical dashed lines shown in the graphs indicate the minimal irradiation doses that were required to achieve 3 log_10_ reduction of CFU counts of the tested bacterial biofilm. +RF +440 nm: irradiated using RF, -RF +440 nm: irradiated not using RF, +Chl +402 nm: irradiated using Chl, -Chl +402 nm: irradiated not using Chl; ns – non-significant. Error bars of some points are too small to be visible.

The minimal required doses of irradiation to achieve ≥3 log_10_ CFU reduction of *Brevundimonas* sp. biofilm ESA1 by RF-API and Chl-API were determined to be 138 J/cm^2^ and 67,5 J/cm^2^, respectively (**Figures 5A, B**). Overall, API studies revealed that *Brevundimonas* sp. ESA1 in its sessile and planktonic growth modes is more sensitive to 402 nm light in combination with Chl.

During RF-API and Chl-API, respective samples of dark controls were collected and analyzed by determining CFU. Respective dark controls with and without PSs (RF or Chl) during the time of its incubation parallel to the illumination experiments did not change significantly and fluctuated within appropriate limits, as well (**Table 3**).

**Table 3:** CFU counts of dark controls (reported in log_10_ scale) of the biofilm.

	Sessile (biofilm) growth mode										
		-RF-440 nm^**^ (CFU/mL)				+RF-440 nm^***^ (CFU/mL)					
Incubation time, min	30	60	90	120	30	60	90		120		
	8.2 ± 0.1*	8.2 ± 0.1*	8.0 ± 0.3*	8.0 ± 0.3*	8.3 ± 0.14*	8.0 ± 0.4*	8.0 ± 0.3*		8.0 ± 0.3*		
	-Chl-402 nm^*^ (CFU/mL)				*-hV* +Chl nm^**^ (CFU/mL)					
Incubation time, min	30	60	90	120	30	60	90	120		
	8.1 ± 0.1*	7.3 ± 0.3*	7.2 ± 0.2*	n	7.8 ± 0.2*	7.7 ± 0.3*	7.8 ± 0.2*	n

* - +RF+440 nm vs -RF-440 and +RF-440 nm or +Chl+402 nm vs –Chl-402 nm and +Chl-402 nm ANOVA test p value <0.05,

** - dark control without RF or Chl, respectively; *** - dark control with RF or Chl, respectively; n – was not evaluated.

It was also determined that effective inactivation of *Brevundimonas* sp. ESA1 biofilms using 402 nm light irradiation without Chl can also be achieved. Although, to reach the minimal 3 log_10_ reduction, a higher dose of ~95 J/cm^2^ was required compared to Chl-based photoinactivation (**Figure 5B**).

For a better understanding of the intrinsic sensitivity of the *Brevundimonas* sp. ESA1 biofilms to the 402 nm light, high irradiation doses were also applied on the planktonic state cells. Results showed that planktonic cells of *Brevundimonas* sp. ESA1 are also sensitive to 402 nm light irradiation without using appropriate PS. Compared to biofilm, ~60 J/cm^2^ dose of 402 nm light was needed to achieve ≥3 log_10_ reduction of CFU counts. Either way, the photoinactivation of *Brevundimonas* sp. ESA1 biofilms solely by 402 nm light need to be studied in more detail to make unquestionable conclusions about the reasons and internal mechanisms of such sensitivity. Nevertheless, both planktonic and biofilm states *Brevundimonas* sp. ESA1 were generally determined to be more effectively killed by light (both 402 and 440 nm) in combination with the corresponding Chl and RF PSs used in this work. In addition, compared to the use of light only, using light in combination with PSs ensures that bacteria will not develop resistance to this antibacterial technology.

#### 3.2.2 Photoinactivation using constant 450 nm irradiation plate

One of the possibilities to practically apply 402 or 440 nm illumination (in hospitals, food settings or spacecraft) to ensure API is to increase the blue part of the general illumination light sources. However, general illumination light sources (even if re-worked to have API function) cannot provide such high irradiance on a wide surface, e.g., 5 mW/cm^2^ and higher values that were used in this work to have a bactericidal effect on planktonic and sessile bacterial cells. Therefore, irradiation for more than 24 h to achieve the desired illumination doses should be considered. To test the long-term illumination, an experiment using constant lower irradiance (1.4 mW/cm^2^) plate emitting only 450 nm blue light was used (**Figure 2A**). Such a “Royal blue” color illumination spectra was chosen since it is widely used in LED-based illuminations systems from plant or aquarium illumination, color lighting to general lighting (part of white light). Furthermore, modern LEDs are achieving extraordinarily high efficiency compared to other light sources. In the current study, only *Brevundimonas* sp. ESA1 biofilm was subjected to a long-term API using a CIP.

**Figure 6:**
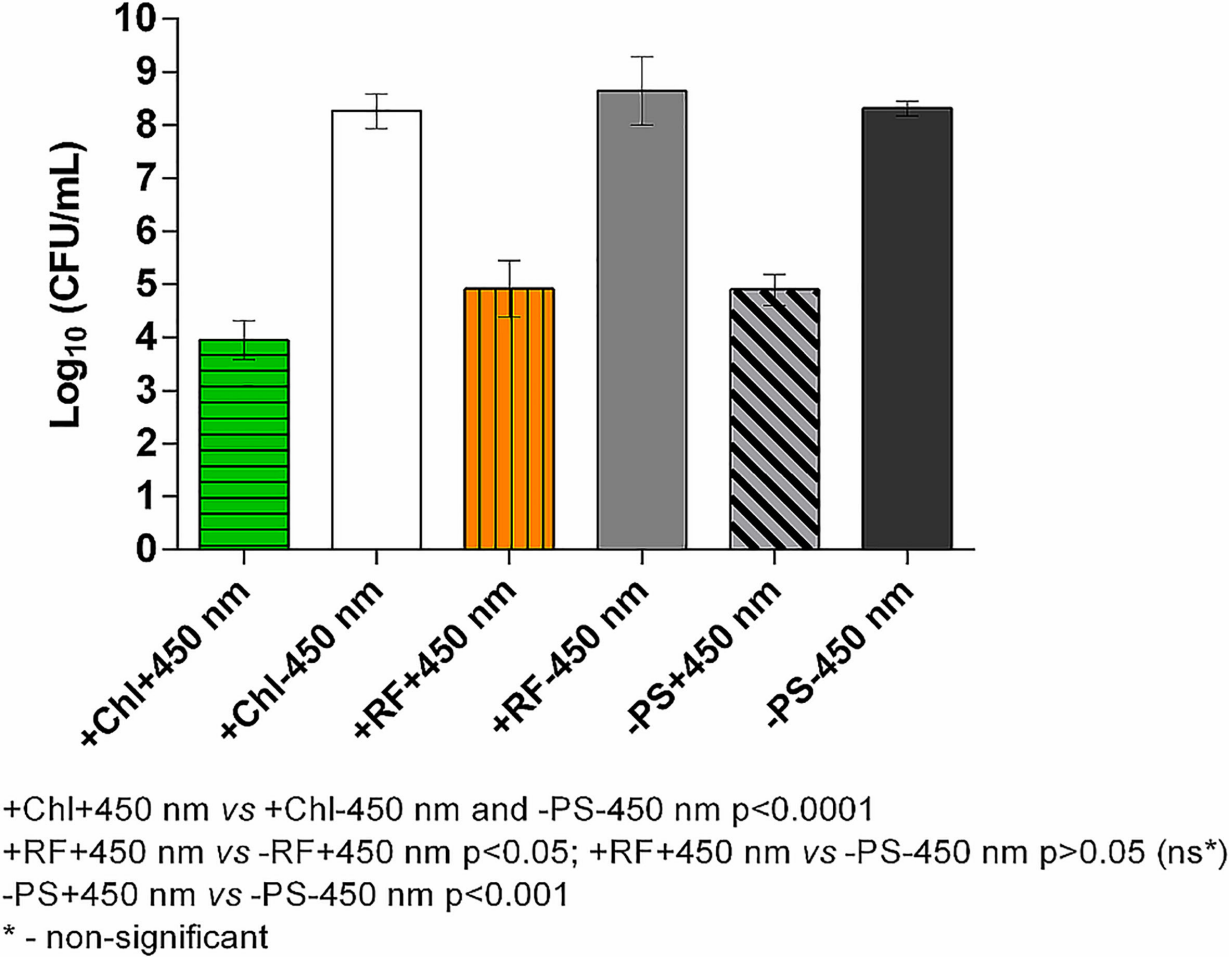
Effect of long-term Chl- and RF-API using CIP at 1.4 mW/cm^2^ (450 nm). +Chl+450 nm/-Chl+450 nm – irradiated 20 h with and without Chl, respectively; +RF+450 nm/-RF+450 nm – irradiated 28 h with and without RF, respectively; -PS+450 nm/-PS-450 nm – irradiated and not irradiated 20-28 h without PSs (Chl or RF), respectively.

Results of API of *Brevundimonas* sp. ESA1 biofilm using CIP at 1.4 mW/cm^2^ showed that long illumination using lower irradiance in combination with either RF or Chl or even without any PSs could be highly effective. For RF-API, irradiation time of 28 h using CIP was equal to a dose of 141.1 J/cm^2^, and for Chl-API, irradiation time for 20 h ensured a dose of 100.8 J/cm^2^ (**Figure 6**). Such long-term irradiation time were used for testing since it was determined that RF-API and Chl-API doses of 138 J/cm^2^ and 67.5 J/cm^2^, respectively, ensure ≥3 log_10_ reduction of CFU when using LED irradiation boxes (**Figures 5A, B**). Long-term Chl-API resulted in >4.3 log_10_ CFU count reduction of the biofilm (**Figure 6**), although 450 nm irradiance is not optimal for the photoexcitation of Chl. In the previous experiment using the irradiation box emitting 402 nm at 20 mW/cm^2^ in combination with Chl, a dose of 67,5 J/cm^2^ was needed to achieve a minimal 3 log_10_ reduction of CFU count of *Brevundimonas* sp. ESA1 biofilm (**Figure 5B**). Long-term Chl-API irradiation time for the achievement of minimal 3 log_10_ reduction can be probably reduced.

Long-term RF-API resulted in ≥3.3 log_10_ reductions of CFU counts of the biofilm (**Figure 6**). The latter result corresponds to the one obtained using the irradiation box (440 nm at 25 mW/cm^2^ dose 138 J/cm^2^) (**Figure 5A**). Long-term illumination of the biofilm without PSs resulted in a bactericidal effect (>3 log_10_ CFU reduction) as well. Therefore, long-term irradiation is efficient not only with RF but also Chl, even though 450 nm is not optimal for the photoexcitation of Chl. Interestingly, long-term blue light (450 nm) irradiation used without PSs is also highly effective in killing *Brevundimonas* sp. ESA1 biofilm caused ≥3.4 log_10_ CFU count reduction of the biofilm.

### 3.3 Biofilm viability study using alamarBlue™

*Brevundimonas* sp. ESA1 biofilm viability after the RF-API and Chl-API (using the irradiation box and irradiation plate) was also investigated by analyzing biofilm-forming cells’ ability to reduce resazurin-based dye alamarBlue™. The latter helps monitor the living cell’s reducing environment and quantitatively measure its viability changes. The dye is water-soluble, non-toxic and permeates cell membranes easily; it is stable (even in culture media) and, therefore, long monitoring is possible. The main active component of alamarBlue™ – resazurin is a redox indicator and has an oxidation-reduction potential (E_0_) of +380 mV at 25°C, pH 7. In a living cell, resazurin (oxidized form) that is blue and non-fluorescent can be reduced in multiple metabolic reactions (by NADPH (E_0_ = 320 mV), FADH (E_0_ = 220 mV), dihydrolipoamine dehydrogenases, NAD(P)H:quinoneoxidoreductases, etc.) to resofurin (reduced state) which has pink color and is highly fluorescent. The change from oxidized to reduced state allows quantitative colorimetric and fluorometric measurements, which in turn allow to determine cell health and viability (Rampersad, 2012; Bonnier et al., 2015). In this study alamarBlue™ changes from reduced to the oxidized state were measured by determining changes in fluorescence.

Viability determination *via* testing of photoinactivated *Brevundimonas* sp. ESA1 biofilm’s ability to reduce alamarBlue^TM^ showed that 3 log_10_ CFU count reduction causing RF-API (irradiation dose ~138 J/cm^2^) and Chl-API (irradiation dose ~67.5 J/cm^2^) induced cellular changes that lead to the inability to reduce alamarBlue™. Such findings approve previously achieved results that *Brevundimonas* sp. ESA1 biofilm after the RF- and Chl-API suffers significant viability loss.

**Figure 7:**
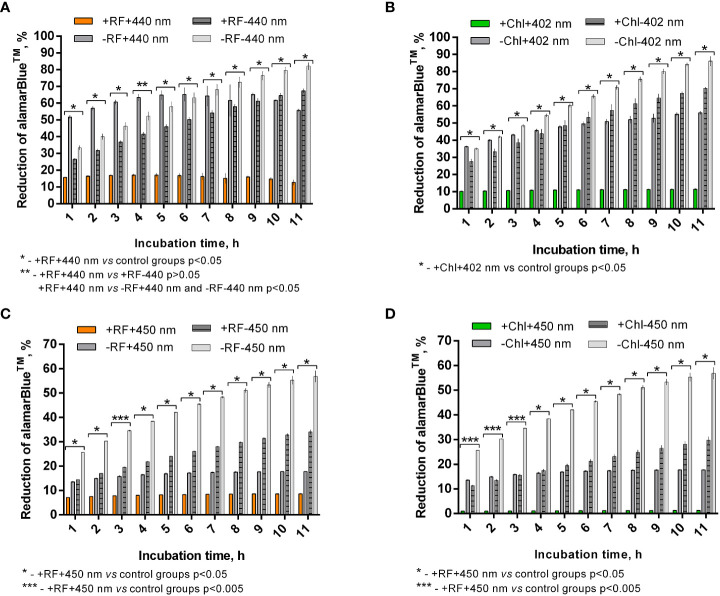
*Brevundimonas* sp. ESA1 biofilm ability to reduce alamarBlue™ after API: **(A)** - RF-API (440 nm at 25 mW/cm^2^ until dose of 138 J/cm^2^ was reached) and **(B)** – after Chl-API (402 nm at 20 mW/cm^2^ until dose 67.5 J/cm^2^ was reached) using the irradiation box; **(C)** - after RF-API (450 nm at 1.4 mW/cm^2^ until dose of 141.1 J/cm^2^ was reached) and **(D)** - Chl-API (450 nm at 1.4 mW/cm^2^ until the dose of 100.8 J/cm^2^ was reached) using irradiation plate. Described irradiation doses ensured the reduction of ≥3 log_10_ in CFU counts. Designations: A, B - +RF+440 nm/-RF+440 nm, +Chl+402 nm/-Chl+402 nm: biofilms irradiated by 440 nm or 402 nm with/without 0.011 mM RF or 0.015 mM Chl, respectively; +RF-440 nm/-RF-440 nm, +Chl-402 nm/-Chl-402 nm: dark controls where biofilms were and were not, respectively, incubated with 0.011 mM RF or 0.015 mM Chl; C, D - +RF-450 nm/-RF-450 nm, +Chl-450 nm/-Chl-450 nm: dark controls of long-term irradiation experiment where biofilms were and were not, respectively, incubated with 0.011 mM RF or 0.015 mM Chl. In cases where irradiation or incubation without PSs was performed, 0.01 M PBS (pH 7.4) was used.

Calculated percentage reduction of alamarBlue™ during the recorded time of incubation of appropriately photoinactivated biofilm, and the dye did not reach more than 15% (**Figure 7A**) and 10% (**Figure 7B**) after RF-API (+RF+440 nm) and Chl-API (+Chl+402 nm), respectively. The biofilm irradiated with 440 nm light without RF (-RF+440 nm) could reduce alamarBlue™, and the ability grew during the incubation time until 8-9 h. At the later time point, percentage reduction reached ~68%; however, subsequent decline was further determined. Dark control biofilm that was incubated with RF (+RF-440 nm) showed an upward dye percentage reduction ability trend with a maximum of ~70% reached at 10-11 h. The biofilm that was incubated in the dark without RF (PBS buffer was used instead) showed increasing alamarBlue™ percent reduction ability during the time of incubation and reached maximum ~85% at 11 h of incubation. Test groups that were irradiated without RF (-RF+440 nm) and incubated (dark control) with RF (+RF-440 nm) had a rather strong ability to reduce the alamarBlue™, which means that the mentioned conditions of treatment of biofilms did not significantly affect the viability (**Figure 7A**). Test groups of biofilms that were irradiated without Chl (-Chl+402 nm) or incubated in the dark with (+Chl-402 nm) or without Chl (-Chl-402 nm), exhibited similar alamarBlue™ percentage reduction values (**Figure 7B**) as it was determined after RF-API (**Figure 7A**).

In general, viability testing with alamarBlue™ after RF-API and Chl-API (using the irradiation box) confirmed the results obtained by determining CFU counts of surviving cells.

After the photoinactivation using 450 nm CIP at 1.4 mW/cm^2^ in combination with RF and Chl, the biofilm of *Brevundimonas* sp. ESA1 exhibited, respectively, only less than 10% or no alamarBlue™ reduction activity. Such results indicate that irradiation using a low irradiance intensity plate for a long period of time caused a significant or total viability loss when using RF or Chl, respectively, compared to AFI using the irradiation box. The control biofilm group that was irradiated with 450 nm without RF or Chl (PBS buffer was used instead of PSs) showed the ability to reduce no more than 20% of alamarBlue™ during all the incubation period (**Figures 7C, D**). Dark control groups, where biofilm was incubated with RF or Chl (groups +RF-450 nm and +Chl-450 nm, respectively) in the dark for an amount of time corresponding to the irradiation time that was used to cause ≥3 log_10_ CFU reduction, showed a low percentage ability of alamarBlue™ reduction. The percentage reduction of the latter tended to grow; however, in the last 4 h of incubation of biofilms with the alamarBlue™ it reached maximal values that were ~60% (**Figures 7C, D**). In comparison to +RF-440 nm, +Chl-402 nm and -RF-440 nm, -Chl-402 nm groups that were incubated for a shorter time using the irradiation box, +RF-450 nm, +Chl-450 nm and -RF/-Chl-450 nm after the long dark incubation showed ~20% lower ability to reduce alamarBlue™. The prolonged incubation that was carried out in the study with CIP could itself have a negative effect on reduction capability that reflects the viability of the biofilm. A negative effect on cell viability could have occurred due to the lack of nutrients during the long incubation time.

### 3.4 Regrowth of RF-API and Chl-API treated biofilm-forming cells

The ability of the *Brevundimonas* sp. ESA1 biofilm to resuscitate and grow in the planktonic state in nutrient-rich liquid LB media (in which planktonic cells for biofilm formation were routinely grown) was lost following irradiation with 450 nm at 1.4 mW/cm^2^ for 20 (Chl-API) and 28 h (RF-API). No changes in OD_600_ of the +RF+450 nm and +Chl+450 nm groups were detected for 72 h (**Table 4**).

**Table 4:** Ability of the biofilm-forming *Brevundimonas* sp. ESA1 cells to regrow in LB media after long-term RF-API and Chl-API (+RF+450 nm and +Chl+450 nm, respectively) using CIP, irradiation without PSs (-PSs(+PBS)+450 nm).

Test group name	OD_600_ change in time		
	24 h	48 h	72 h
+RF+450 nm	0	0	0
+Chl+450 nm	0.05 ± 0.01	0	0
-PS(+PBS)+450 nm	0	0.01 ± 0.01	1.96 ± 0.02
+RF-450 nm	0.71 ± 0.4	1.91 ± 0.04	3.08 ± 0.34
+Chl-450 nm	1.09 ± 0.09	1.82 ± 0.11	2.72 ± 0.51
-PSs(+PBS)-450 nm	0.78 ± 0.57	1.78 ± 0.1	2.87 ± 0.29

Respective dark controls incubated with or without RF or Chl (+RF-450 nm, +Chl-450 nm and -PSs(+PBS)-450 nm are also indicated in the table.

Regrowth analysis of the biofilm test group, which was illuminated with 450 nm without PSs (-PS+450 nm) showed that for the first 48 h the growth did not occur; however, on the third day (in 72 h) OD_600_ reached 1.96 ± 0.02. Irradiation with 450 nm without PSs delayed regrowth for 2 days. All the tested dark controls (+RF-450 nm, +Chl-450 nm, -PSs(+PBS)-450 nm) demonstrated the ability to regrow (**Table 4**).

Regrowth of *Brevundimonas* sp. ESA1 biofilm following 3 log_10_ CFU reduction causing RF- and Chl-API using the irradiation box did not occur for 48 h (OD_600_ = 0) but bacteria visibly resuscitated on the third day of cultivation in LB with OD_600_ values reaching 3.55 ± 0.07. Biofilms that were illuminated either with 402 or 440 nm regrew up to OD_600_ values equal to 3.5 ± 0.70 and 3.65 ± 0.91, respectively, on the second day of growth measurement. Illumination in combination with PSs and sole illumination delayed the regrowth of *Brevundimonas* sp. ESA1 for 2 and 1 day, respectively. Biofilms that formed dark control groups did not have such lag phases. OD_600_ changes of all tested biofilm groups after the photoinactivation using the irradiation box are shown in **Table 5**.

**Table 5:** Ability of the biofilm-forming *Brevundimonas* sp. ESA1 cells to regrow in LB media after short-term RF- and Chl-API (+RF+440 nm and +Chl+402 nm, respectively) using the irradiation box, irradiation without PSs (-RF+440 nm and -Chl+402 nm).

Test group name	OD_600_ change in time		
	24 h	48 h	72 h
+RF+440 nm	0.005 ± 0.01	0.015 ± 0.021	3.55 ± 0.07
-RF+440 nm	0.005 ± 0.01	3.65 ± 0.91	4.25 ± 0.55
-RF-440 nm	2.25 ± 0.55	3.75 ± 0.9	3.75 ± 0.52
-RF-440 nm	2.14 ± 0.65	3.5 ± 0.70	3.84 ± 0.62
+Chl+402 nm	0.01	0.01 ± 0.014	3.52 ± 0.11
-Chl+402 nm	0.015 ± 0.01	3.5 ± 0.70	4.07 ± 0.86
+Chl-402 nm	2.16 ± 0.62	3.9 ± 0.69	3.61 ± 0.6
-Chl-402 nm	2.95 ± 0.0.64	4.15 ± 0.91	3.8 ± 0.57

Respective dark controls incubated with or without RF or Chl (+RF-440 nm, -RF-440 nm and +Chl-402 nm, -Chl-402 nm) are also indicated in the table.

The studies of the ability of the biofilm cells to regrow in suspension and previous viability testing experiments *via* evaluation of alamarBlue™ reduction after the RF- and Chl-API have revealed that, apparently, some cells after the RF-, Chl-API or irradiation without PSs enter viable but non-culturable state (VNBC). Such a conclusion was made since some photoinactivated biofilm test groups were metabolically active (reduced alamarBlue™) but at the same time did not grow in the liquid nutritionally rich LB medium. The biofilm that was exposed to 450 nm light (test group +450 nm -RF/-Chl (+PBS)) was metabolically active and reduced alamarBlue™ for more than 11 h up to ~20% (**Figure 7C**). However, its OD_600_ values over 48 h were equal to 0 with subsequent growth detection at 72 h (**Table 4**). Such results indicate that only a part of the cells entered a VBNC state which was a cause of bacterial resuscitation, while most biofilm cells did not survive after exposure. VBNC state was also induced in *Brevundimonas* sp. ESA1 biofilm cells were exposed to RF-API (+RF+440 nm test group) and Chl-API (+Chl+402 nm test group) using the irradiation box. Biofilms of these test groups after appropriate PS-based photoinactivations could reduce alamarBlue™ up to ~60-70% at 12-14 h incubation points (**Figures 7A, B**), but at the same time cell growth in LB media was not detected for 48 h. Subsequently, bacteria resuscitated only on the third day of growth, reaching OD_600_ corresponding to those measured for the dark controls (**Table 5**), meaning that most of the bacterial cell population transitioned to VBNC state. Biofilm cells that were exposed to 402 or 440 nm irradiation without PS, recovered faster than those that were subjected to photoinactivation in combination with PSs (**Table 5**).

### 3.5 Scanning electron microscopy

**Figure 8:**
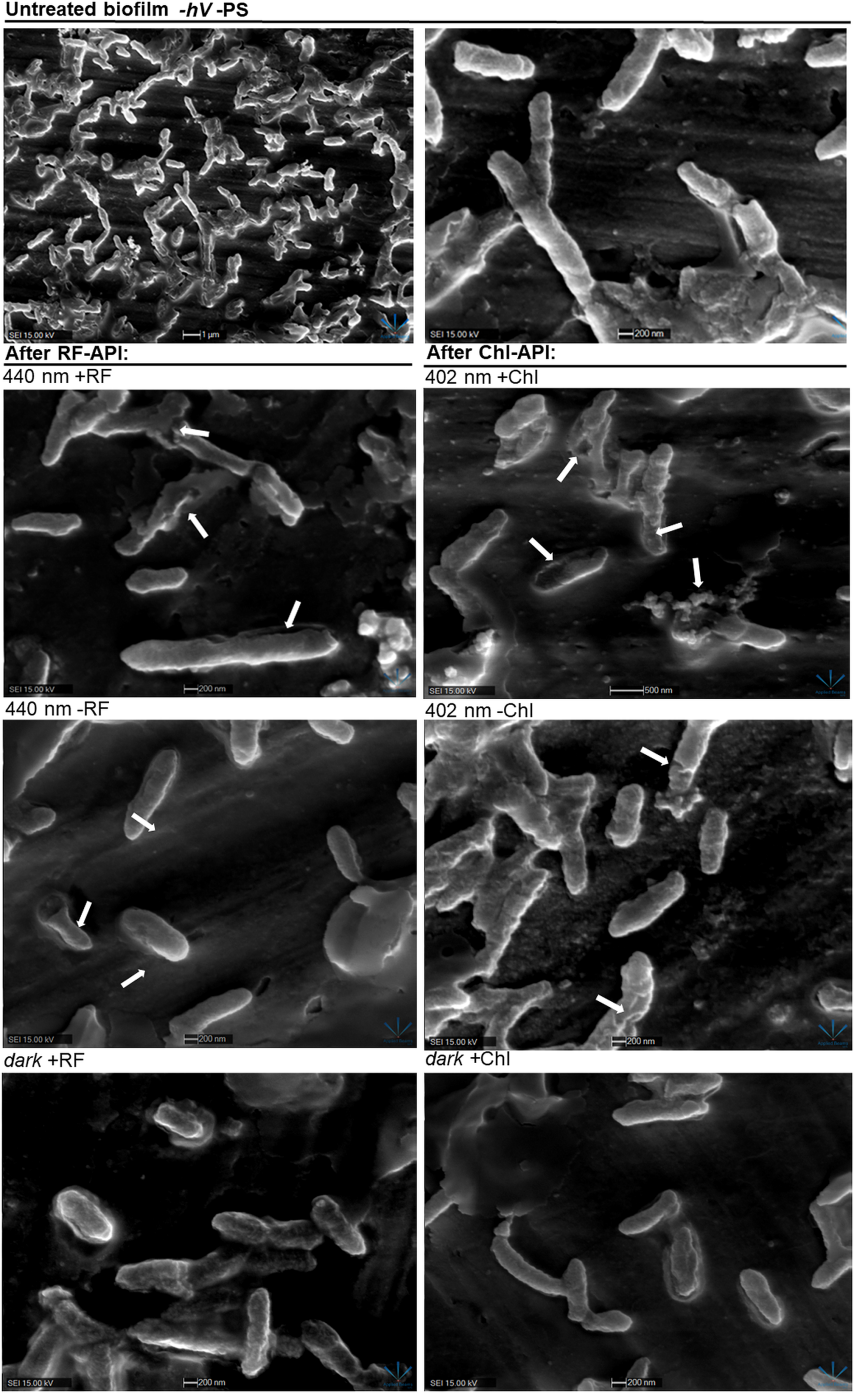
SEM images of *Brevundimonas* sp. ESA1 biofilm cells: *untreated (-hV* –PS: no PSs (RF nor Chl) in dark conditions); after *RF-API*: 440 nm +RF (treated with 0.011 mM RF and irradiated by 440 nm up to 140 J/cm^2^), 440 nm -RF (not treated with 0.011 mM RF prior to irradiation, cells were covered with 0.1 M phosphate buffer (pH 7.4) and irradiated by 440 nm up to 140 J/cm^2^), *dark* +RF (biofilms treated with 0.011 mM RF and incubated in the dark for an amount of time corresponding to time needed to achieve 140 J/cm^2^); after *Chl-API*: 402 nm +Chl (treated with 0,015 mM Chl and irradiated by 402 nm up to 70 J/cm^2^), 402 nm -Chl (not treated with 0,015 mM Chl prior to irradiation, cells were covered with 0.1 M phosphate buffer (pH 7.4) and irradiated by 402 nm up to 70 J/cm^2^), *dark* +Chl (biofilms treated with 0,015 mM M Chl and incubated in the dark for an amount of time corresponding to time needed to achieve 70 J/cm^2^).

Scanning electron microscopy revealed that RF-API and Chl-API using the irradiation box at doses of 138 J/cm^2^ and ~70 J/cm^2^, respectively, have induced changes in biofilm cell morphology of investigated bacteria compared to untreated cells (**Figure 8**).

After both RF-API and Chl-API, mechanically detached biofilm cells appeared to have disrupted and perforated cell walls, and some appeared lysed. Significant morphological changes were also seen after irradiation with 402 nm without using Chl. That is a logical outcome since CFU count analysis after irradiation has shown that 402 nm light without PS can have a reducing effect on viability as well (**Figure 5B**). Cells of the dark controls did not have any noticeable damage to the cell walls, but the surface of those incubated in the dark with RF did not look as smooth as after incubation with Chl.

## 4 Discussion

### 4.1 Photoinactivation of *Brevudimonas* sp. ESA1

This study was the first to demonstrate the efficiency of the RF- and Chl-mediated photoinactivation of *Brevundimonas* sp. ESA1 biofilms. In particular, it was shown that Chl-based API required half the irradiation dose compared to RF-API. Therefore, Chl-API can be considered a more effective method for the inactivation of studied biofilms and planktonic cells. Moreover, it was determined that effective inactivation of *Brevundimonas* sp. ESA1 biofilms using 402 nm light irradiation without Chl can also be achieved. Although to reach the minimal 3 log_10_ reductions, a higher dose was required compared to Chl-based photoinactivation (95 J/cm^2^ instead of 67.5 J/cm^2^). Such phenomenon has not been observed after RF-API and Chl-API of *Brevundimonas* sp. ESA1 planktonic cells in the used dosage range of the photoinactivation. The use of PS, in the case of Chl-API of *Brevundimonas* sp. ESA1 biofilm can be considered redundant/unnecessary to achieve inactivation (bactericidal or bacteriostatic effect). The fact that biofilms can be sensitive to light without PSs can have practical significance in facilitating the application of the technology in systems where spreading PSs can be complicated, e.g., in a spacecraft environment, where microgravity conditions prevail. However, the cause of *Brevundimonas* sp. ESA1 to be sensitive, particularly in its biofilm form to 402 nm only (without Chl) is currently unknown. In general, the sensitivity of biofilms to blue light can be explained by the fact that bacteria can have endogenous porphyrins that can be sensitized by 402 nm and can generate ROS, resulting in cellular photo-oxidative reactions that initiate various cellular damages and death; the light can also activate prophages that cause bacterial cell lysis (Ferrer-Espada et al., 2019). A few studies have shown that biofilms of different Gram-negative (*Salmonella*, *Escherichia coli* O157:H7, etc.) bacteria can be equally sensitive or even more sensitive to irradiation compared to planktonic cells (Niemira and Solomon, 2005; Niemira, 2007; Niemira, 2010). The sensitivity to API of the biofilm and the planktonic cells of bacteria can be different and depend on the metabolic states and the growth phase as well (Blee et al., 2020). It can also be assumed that the cause of the higher sensitivity of the *Brevundimonas* sp. ESA1 biofilm to the 402 nm light is somehow associated with its extracellular polymeric matrix (Flemming and Wingender, 2010). Although, EPS is usually the cause of the higher doses needed to inactivate bacteria in sessile form compared to planktonic cells. For example, this study’s experimental results have shown that about 6 times higher irradiation doses are needed to achieve the bactericidal effect on biofilms compared to planktonic cells. Therefore, the improvement of API technology for the more effective killing of bacterial biofilms should be certainly considered. The efficiency of API can be improved by testing higher concentrations of PSs, a step of preincubation with PSs before irradiation and synergy studies. Additionally, using antimicrobials can be done as well.

API is a two-stage technology consisting of the natural PS, which must be applied on a surface, and an illumination system providing a sufficient dose of light to inactivate bacteria. This makes it complicated, especially for the application in microgravity conditions, and requires a well-developed application plan as well as proper engineering solutions. Reworking of currently installed lighting fixtures (e.g. SSLAs on ISS), increasing the blue part of the general lightning, and long irradiation time could be one of the options. Based on this assumption, to test the long-term illumination, an experiment using constant lower irradiance (1.4 mW/cm^2^) plate emitting only 450 nm blue light was used (**Figure 2A**). Such color illumination spectrum was chosen since it is widely used in LED-based systems such as general lighting (part of white light).

Results of API of *Brevundimonas* sp. ESA1 biofilm using CIP at 1.4 mW/cm^2^ showed that long illumination using lower irradiance in combination with either RF or Chl or even without any PSs could be highly effective. However, some additional long-term irradiation experiments using CIP can be performed to evaluate the 450 nm effect in combination with RF, Chl or without the use of PSs, and if the internal porphyrins might have had a significance for the API of the *Brevundimonas* sp. ESA1. The latter phenomenon has already attracted increasing attention and several studies have shown that blue light, particularly in the wavelength range of 405–470 nm has intrinsic antimicrobial effect on different microbes including Gram-positive and Gram-negative bacteria without the addition of exogenous PSs (Dai et al., 2012; Hoenes et al., 2018; Gwynne and Gallagher, 2018: Hadi et al., 2020). Nevertheless, long-term irradiation indeed showed to be effective and promising for practical implementation of API customization. However, reworking installed lighting fixtures or sending additional API-dedicated illumination to spacecraft definitely results in extra expenses that must be evaluated and weighted in detail. On the other hand, when developing new modules for ISS or other space missions, the API could be considered an antimicrobial technique. Therefore, if performed on earth, the integration of API illumination systems would have a neglectable effect on the total costs and weight of the project.

### 4.2 Transition of *Brevundimonas* sp. ESA1 to VBNC state

Viability testing with alamarBlue™ after RF-API and Chl-API (using the irradiation box and CIP) confirmed the results obtained by determining CFU counts of surviving cells. However, alamarBlue™ reduction studies in addition to bacterial regrowth studies, had shown that *Brevundimonas* sp. ESA1 biofilm cells, after the application of RF-API and Chl-API, enter VBNC state.

Only a few published studies announce the transition of certain bacterial species to the VBNC state after exposure to blue light or violet-blue lights (Abana et al., 2017; Hoenes et al., 2018).

In general, VBNC state is characteristic to non-spore-forming, usually Gram-negative bacteria and appears under various stressful conditions. VBNC cells may be resuscitated back to cultivable cells under suitable stimuli. The conditions that trigger the induction of the VBNC state and resuscitation can be different for different bacteria species, e.g., pathogens usually resuscitate only *in vivo* (Li et al., 2014; Zhang et al., 2021). This study was the first to demonstrate that bacteria belonging to the genus *Brevundimonas* sp. ESA1 can enter the VBNC state after its photoinactivation and in some cases resuscitate in nutrient-rich conditions. Moreover, undesired growth after the long-term RF- and Chl-API is unlikely to occur even in nutrient-rich environments.

### 4.3 Guidelines for further research and development of API in space conditions

Despite the efficiency of Chl- and RF-based API against *Brevundimonas* sp. ESA1 and other bacteria (that was demonstrated elsewhere), use of the PSs have a huge potential for improvement. For example, the mixes of PSs and the combinations of PSs with polymers and other materials can be investigated. Moreover, an in-depth investigation of the long-term, low-irradiance API technologies could help find photoinactivation solutions under general or natural lighting conditions.

Design and development of the API PS solution already integrated into the newly developed space premises and/or modules should be considered in order to apply the technology conveniently in conditions of low gravity. If the integration of the PSs is not possible, investigation and design of API application techniques (e.g. sprinklers) in microgravity conditions must be considered as well.

## 5 Conclusions

API based on natural PSs RF or Chl and illumination by blue light (402-450 nm) has the potential to be applied as an antimicrobial technique against planktonic and, most importantly, more recalcitrant biofilm form of *Brevundimonas* sp. ESA1. Recently, some species of the latter bacterial genus have been revealed as a global opportunistic pathogen that can be dangerous for individuals with chronic underlying diseases and immunocompromised people. *Brevundimonas* spp. are abundant in terrestrial and even confined spacecraft environments.

The main advantage of the RF- and Chl-mediated API compared to other antimicrobial methods is that this technology is non-toxic and could be safely used in closed-loop, food, drinking water, and other systems as an antimicrobial technology posing no risk to humans or other living creatures. Moreover, there has been only a neglectable or no resistance development towards API observed since the beginning of its use. Implementing the API technology in the confined systems as spacecraft, its application could consist of three steps, in particular, preparation of the aqueous solutions of the PSs, application of the PSs on the target surface, and irradiation of that surface by the required light spectrum and sufficient light amount (dose).

In this work, by comparing the optical absorption spectra of the PSs and irradiance spectra of LEDs, it was determined that 402 nm and 440 nm are optimal to excite Chl and RF, respectively. Photostability experiments showed the photomodifications of both PSs under the conditions subsequently used to inactivate *Brevundimonas* sp. ESA1. Besides the demonstration of the RF-based and Chl-based API conditions needed to inactivate *Brevundimonas* biofilms using two different illumination systems, a study of the API treated bacteria to reduce alamarBlue™ and regrowth studies revealed that *Brevundimonas* sp. ESA1 enters VNBC state without resuscitation after long-term irradiation in conditions tested in this work.

Nevertheless, further investigation of the API applicability should be carried out, focusing on certain application areas such as HVAC, water supply, sanitary systems, plant growth facilities, etc.

## Data availability statement

The data presented in the study are deposited in the NCBI GenBank repository, accession number ON237360; https://www.ncbi.nlm.nih.gov/nuccore/ON237360.1/.

## Author contributions

AG and IB, conceptualization, methodology, and data processing. AG, IB, and KB, experiment implementation. AG, formal analysis, writing – original draft, writing – review and editing, visualization. IB, LK, and PV, writing – original draft, writing – review and editing. All authors contributed to the article and approved the submitted version.

## Funding

Part of the research was funded by the European Space Agency (LT5_1 ESA Contract No. 40000129495/19/NL/SSC).

## Acknowledgments

Authors would like to acknowledge the European Space Agency (ESA).

## Conflict of interest

The authors declare that the research was conducted in the absence of any commercial or financial relationships that could be construed as a potential conflict of interest.

## Publisher’s note

All claims expressed in this article are solely those of the authors and do not necessarily represent those of their affiliated organizations, or those of the publisher, the editors and the reviewers. Any product that may be evaluated in this article, or claim that may be made by its manufacturer, is not guaranteed or endorsed by the publisher.
